# A Systematic Review on the Effectiveness of Machine Learning in the Detection of Atrial Fibrillation

**DOI:** 10.2174/011573403X293703240715104503

**Published:** 2024-07-31

**Authors:** Lubabat Wuraola Abdulraheem, Baraah Al-dwa, Dmitry Shchekochikhin, Daria Gognieva, Petr Chomakhidze, Natalia Kuznetsova, Philipp Kopylov, Afina Avtandilovna Bestavashvilli

**Affiliations:** 1 World-Class Research Center «Digital Biodesign and Personalized Healthcare», I.M. Sechenov First Moscow State Medical University (Sechenov University), 119991 Moscow, Russia

**Keywords:** Atrial fibrillation, ECG, artificial intelligence, machine learning, arrhythmia, neural networks

## Abstract

Recent endeavors have led to the exploration of Machine Learning (ML) to enhance the detection and accurate diagnosis of heart pathologies. This is due to the growing need to improve efficiency in diagnostics and hasten the process of delivering treatment. Several institutions have actively assessed the possibility of creating algorithms for advancing our understanding of atrial fibrillation (AF), a common form of sustained arrhythmia. This means that artificial intelligence is now being used to analyze electrocardiogram (ECG) data. The data is typically extracted from large patient databases and then subsequently used to train and test the algorithm with the help of neural networks. Machine learning has been used to effectively detect atrial fibrillation with more accuracy than clinical experts, and if applied to clinical practice, it will aid in early diagnosis and management of the condition and thus reduce thromboembolic complications of the disease. In this text, a review of the application of machine learning in the analysis and detection of atrial fibrillation, a comparison of the outcomes (sensitivity, specificity, and accuracy), and the framework and methods of the studies conducted have been presented.

## 1 INTRODUCTION

### 1.1 Atrial Fibrillation

It is the most common form of prolonged arrhythmia experienced in the aging population, with an increased risk of thrombo-embolic complications, such as stroke. Moreover, it is also known to result in potentially significant morbidity in patients, particularly stroke, depression, left ventricular dysfunction, and reduced quality of life [1-3]. Therefore, screening for atrial fibrillation is an important aspect of improving the outcome of the disease in the population. It can be symptomatic or asymptomatic and is classified by the European Society of Cardiology (ESC) into the first-diagnosed form, the paroxysmal, persistent, long-standing persistent, and permanent sub-types. Many atrial fibrillation episodes are asymptomatic, and they have been implicated as having an isolated association with increased stroke risk, with a new diagnosis of AF being established in 10% of stroke presentations [4-6].

The condition has also been observed, albeit complex, in associations with other chronic systemic conditions like hypertension, diabetes, coronary heart disease, and cardiac failure [7-10]. The latter especially has been noted in patients with unsatisfactorily controlled forms of arrhythmia. The mainstay of AF management is rate control and anti-coagulation for those symptomatically limited by the disease. In certain patients with symptomatic AF and heart failure with reduced left ventricular ejection fraction, catheter ablation may be suggested to potentially lower the mortality rate and reduce hospitalization rates for cardiac insufficiency [11-14].

A significant amount of AF still goes undiagnosed, with a frequency of 11–13% of the total prevalence, according to the data reviewed from various studies (although higher in countries with high GDP levels). It is of even more clinical concern to note that more than half of the observed population with undiagnosed AF had a moderate or high risk of stroke (most had CHA2DS2-VASc ≥2). This makes it essential that more of these undiagnosed cases are identified and attended to early [15,16]. Some studies have stated that atrial fibrillation was successfully detected while the patient still had sinus rhythm using artificial intelligence-enabled ECG [17,18]. This lends a helping hand to medical staff in choosing the most appropriate management approach, as they can predict which patients will most likely benefit from interventions like cardioversion [19, 20].

Furthermore, a larger proportion of the diagnosed cases of AF proceed to more persistent forms of arrhythmia, and fewer of them are completely arrested in the acute stage. A study assessed 720 known diagnoses of AF and found that less than a fourth of them were acute forms of the disease, which had a male predominance. Over 78% of the patients had chronic forms of the disease, making it an overwhelming majority. Persistent forms of AF are unsurprisingly linked with a higher likelihood of comorbid conditions and elevated risks. According to the same study, women were more likely to have persistent forms of AF, which could result in a predisposition to developing complications, a matter of particular importance as females have a higher risk of bleeding if put under anticoagulation therapy [16, 21-23].

With more patients having persistent forms of the disease, there is an increased need for rhythm-controlling interventions like ablation in patients with clinical symptoms accompanied by co-morbid conditions like cardiac insufficiency. Ablation is a treatment method based on the principle of identifying and terminating foci of arrhythmia and breaking the re-entry chain (*e.g*., for relief of foci of ectopic activity in pulmonary veins in AF) [24, 25]. It is typically indicated for atrial fibrillation with lifestyle-limiting symptoms, intolerance to the anti-arrhythmic agent prescribed, or unsuccessful therapeutic interventions. A 2023 meta-analysis comparing catheter ablation and medical therapy for heart failure patients with AF by Faysal *et al.* supported the significant role catheter ablation plays in the overall reduction of mortality. It has also produced positive results with ejection fraction and the general quality of the patient's life [21, 22] and reduced the stroke risk, mortality rate, and hospitalisation frequency for heart failure (95% CI: 0.43-0.76) [14]. Hybrid approaches could, however, be considered with both surgical and catheter ablation in patients with complex AF, according to other studies [26-29].

The results after ablation are usually favourable; they depend, however, on the form of existing AF and the presence of cardiovascular disorders, but can reach 80-85%. The probability of success may be 30% lower if structural diseases are present (*e.g*., structural heart defects), making it valuable to have an effective means to preselect and filter patients who would most likely need the procedure and actually benefit from earlier treatment. After ablation therapy, artificial intelligence can also be used to estimate the risk of a recurrence of AF [30-32].

Therefore, active efforts need to be made by the medical community and relevant affiliates to swiftly note the patterns of development of atrial fibrillation, detect and accurately predict the dynamics of the condition at an early stage, and manage it accordingly. AI algorithms can use ECG data to detect atrial fibrillation or bradycardia, notify patients to seek medical attention, and track ECG changes during exercise, sleep, or stress tests [33-36].

### 1.2 Machine Learning

It is a sub-field of artificial intelligence and is generally defined as the ability of a machine to imitate intelligent human behavior; it is a system used to perform complex tasks in a similar manner to how humans solve problems. It has been defined as “the field of study that gives computers the ability to learn without explicitly being programmed”. This means that machines can recognize visual representations, understand written texts in natural language, or perform an action in the physical world. It has become one of the most significant ways through which AI has been used in the past decade and is increasingly being applied to medical diagnostics [37], in particular, the interpretation of electrocardiograms and the detection of cardiac abnormalities. A part of machine learning, known as the Deep Neural Network (DNN), plays a substantial role in the development of ML systems. DNN is an ML training model that includes several layers of input data stored in a successive fashion. The result of which will be a system capable of carrying out a targeted task. In general, the function of a machine learning system can be descriptive (explains what happened), predictive (predicts what will happen), or prescriptive (uses the data to make suggestions about what action to take) [38]. For the purpose of creating a machine model that can identify ECG-detectable cardiac pathology, electrocardiograms from a large database are divided into training and testing sets for the purpose of training a computer algorithm to detect them [3, 19, 39, 40].

The purpose of this review is to summarize briefly some of the currently available data on the usage of machine learning in the identification process of AF, to make a comparison of the methods and results of the study, and to highlight the risks and downsides of this process.

## 2 RESEARCH METHODOLOGY

### 2.1 Criteria for Inclusion Were

- Study published within the past 10 years.
- Research conducted using machine learning algorithms.
- A neural network learning model must be included in the framework.
- Patient data were collected from patients aged 18 and older.

Most research was taken from the National Centre for Biochemical Information (NCBI), the PubMed Database, Elsevier, the Lancet journal, and ResearchGate. The search was conducted by a PhD student of cardiology.

In this review, the diagnostic effectiveness of neural networks in atrial fibrillation is investigated by making a comparison between the sensitivity, specificity, and accuracy across various machine learning-based studies, highlighting the differences in the forms of neural networks used. Mostly quantitative data were taken from studies; they included the sizes of databases (number of patients used and ECGs recorded). They also contained the results and data about the sensitivity, specificity, and accuracy of the results that were present. The remaining data was qualitative in nature, including but not limited to the manner of processing data in each neural network, methods of training and testing the system, and limitations encountered. The general information about machine learning was compiled from several scientific sources (*e.g*., research, reviews, *etc*.) over the past two decades.

After the review was completed, it was rechecked by a minimum of 5 different personnel from the Department of Cardiology and School of Digital Medicine, and necessary modifications were made.

**Fig. (1):**
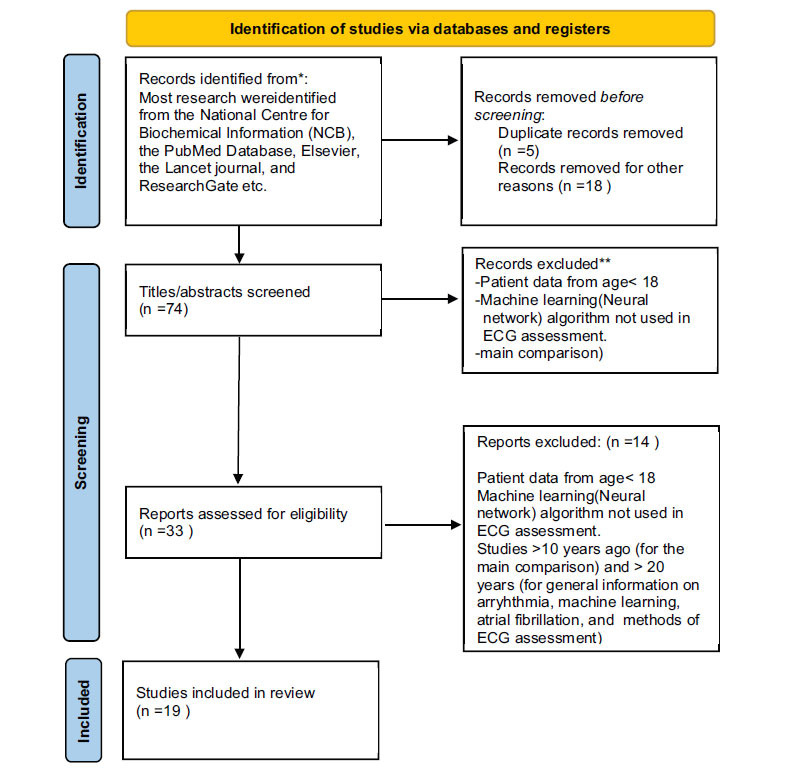
PRISMA 2020 flow diagram.

Relevant parts of the research used in the comparison were tabulated using MS Excel (Fig. **1**).

## 3 OVERVIEW OF MACHINE LEARNING METHODS

Machine learning models fall into a few primary categories (Table **1**).

### 3.1 Supervised Machine Learning

This method is employed under expert surveillance. Using it, the ML model produces desired results using generated output information (of substantial quantity) that has been fine-tuned. The results are subsequently tested until a satisfactory pattern is confirmed, which can then be used for new analysis in the future. This method is frequently applied to classification and regression techniques, neural networks, linear regression, logistic regression, random forest, and support vector machines. It can be feasible in image recognition and, unsurprisingly, in stock pricing.

### 3.2 Unsupervised Learning

As the name implies, it does not need human interference. It uses unlabeled data to train the ML model. Without any specific guide, the model is expected to note tendencies and identify patterns from input data (no output needed). Unsupervised learning can be used in clustering (organization of large datasets), the detection of anomalies, and also for image analysis.

### 3.3 Semi-supervised Learning

It falls between the two other methods. A small data set is used to classify a larger, unlabeled one. This form of ML can help to tackle the problem of not having sufficient labeled data or not having adequate funds to label enough of it for a supervised learning algorithm.

### 3.4 Reinforcement Learning

This form of ML learns from experience and is able to perceive and interpret its environment. It adopts a trial and error pattern to obtain results and then applies it in the future as an improved version without repeating previous mistakes. Unsurprisingly, this method of ML is applied in video gaming.

## 4 CLINICAL APPLICATIONS OF ML-ALGORITHM IN ARRHYTHMIA DETECTION

Following are the clinical applications of ML-algorithm in the detection of arrhythmia:

- Atrial fibrillation event detection and classification.
- Screening for atrial fibrillation.
- Identification and recording of AF episodes on integrated devices (both in-hospital and portable) for subsequent assessment by a specialist to guide clinical decision-making.
- Risk stratification and prediction.
- Regular monitoring and management of patients with atrial fibrillation.

## 5 COMPARISON OF METHODS AND RESULTS OF DIAGNOSTIC MACHINE LEARNING ALGORITHMS IN ARRHYTHMIA DETECTION

Many studies involving the exploration of machine learning for the detection of arrhythmias use deep neural networks or subsets of them, such as Convolutional Neural Networks (CNN). CNN is distinguished from other neural networks by its superior performance with image, speech, or audio signal inputs. It is commonly used for analysis of visual imagery [41]. Another subset of deep learning used is the Deep Belief Network (DBN), a type of deep learning architecture that is a combination of unsupervised networks (such as restricted Boltzmann machines (RBMs) or auto-encoders). DBN was invented as an answer to the problems encountered when using traditional neural network training in deep layered networks, such as slow learning, poor parameter selection, and the requirement of a lot of training datasets. This article presents a summary of studies using different neural networks for diagnostic purposes, highlighting the differences in their methods and the corresponding results.

In 2019, a study conducted at Stanford University employed the use of Deep Neural Networks (DNN) to detect 12 rhythm classes from raw single-lead ECG inputs using a training data set of over 90,000 ECGS (median of 30 seconds each). Ten different forms of arrhythmia were identified and separated from sinus rhythm, and artifact noise was delineated. The ECGs were recorded (single vector style) using an FDA-approved ambulatory monitor, Zio®. The average recording duration was 10.6 days. Patients were randomly sampled [42].

A comparison was made afterward between the results obtained from the DNN and the assessment by a group of certified cardiologists. This was done using the Deep Neural Network algorithm area under the receiver operating characteristic curve, otherwise known as the AUC. To assess accuracy, the cardiologists marked the record (set level) at the start and end of each class of rhythm and labelled the output intervals (sequence level). In all 10 arrhythmias, the AUC was higher than 0.91 and even more so in arrhythmias like atrial fibrillation and tachycardia (both atrioventricular and ventricular tachycardia).

**Fig. (2):**
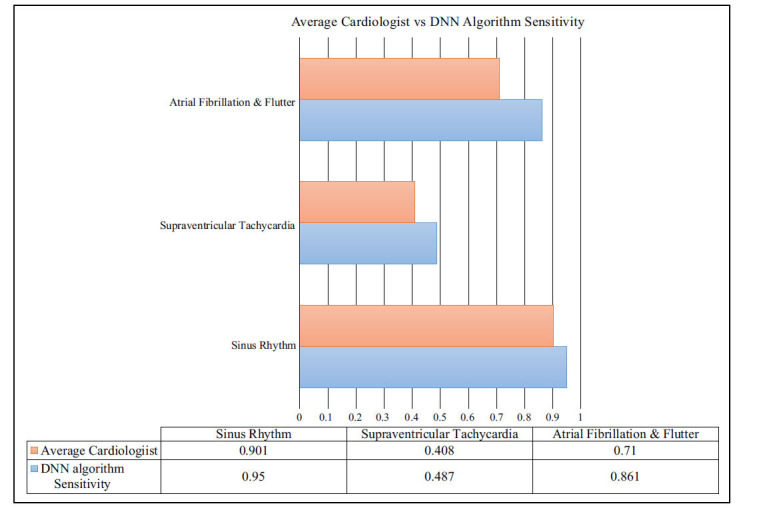
Showing comparison between normal physician and ML algorithm effectiveness.

Following that, other experts individually reassessed the records, and a second comparison was made between their F1 scores and those of the algorithm (the mean of the positive predictive value (precision) and sensitivity (recall)). The results were in favour of the algorithm (the sensitivity of the model was higher while specificity was at a similar level to the one identified by a cardiologist) (Fig. **2**).

However, an earlier study (2017) conducted by Bahareh *et al.* at Carleton University attempted to improve the accuracy of the ECG readings in order to eliminate the likelihood of false positives and, as a result, the highly undesirable false alarms and panic were observed among users of portable devices. They did this by isolating extrinsic factors that typically make portable readings less reliable than bedside electrocardiograms (*e.g*., motion artifacts, powerline interference with the device signal) due to the absence of a well-controlled environment. This was accomplished by the usage of Deep Belief Networks (DBN) (because of their strength in feature extraction and classification) based on a three-layer Restricted Boltzmann Machine (RBM). The first two RBMs were trained to extract the features and apply them to a third layer of RBM that would subsequently classify the data to have an algorithm that would classify measured ECGs based on the quality of the signal, which would differentiate between noisy and clean signal measurements. ECGs with low signal quality could undergo additional pre-processing to mitigate the contaminants, or the signal could simply be discarded [43].

**Fig. (3):**
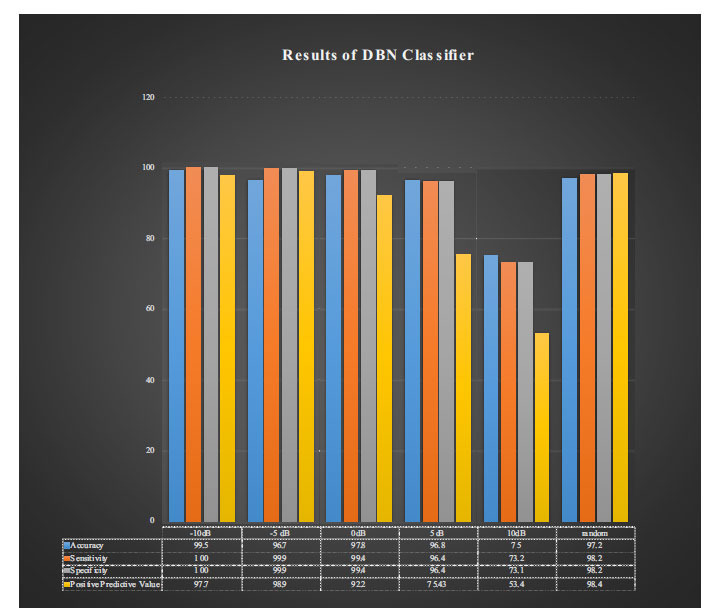
Results of DBN.

**Fig. (4):**
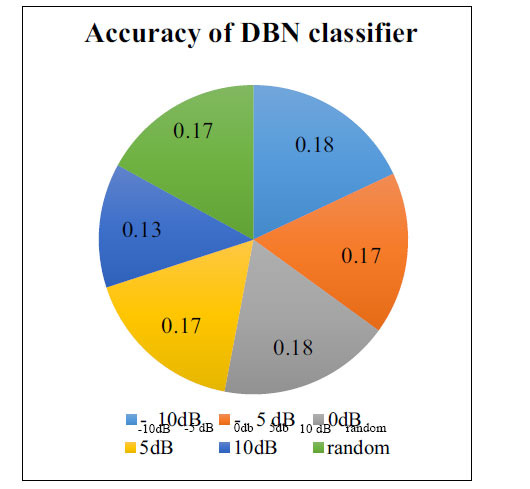
Accuracy of DBN classifier across different sound levels.

Results suggested that the DBN classifier does not get confused and can identify clean segments regardless of whether they are arrhythmic or not. Results were better with lower SNR because noisy signals were easier to discern from clean signals, given the higher level of noise (Figs. **3** and **4**).

In 2021, the Mayo Clinic published research in which they developed an algorithm trained on almost 2.5 million standard 12-lead ECGs from over 720 000 adult patients obtained at the Mayo Clinic ECG laboratory from 2007-2017 that was thereafter compared with a computerized ECG reader (Marquette 12SL)[44]. The process consisted of model development, training (1,749,654), validation (249,951 ECGs), and testing (499,917 ECGs) of the system.

A Convolutional Neural Network (CNN) was used to translate ECG features into 66 ECG codes that could detect various changes, such as cardiac ischemia, arrhythmia, conduction abnormalities, signs of structural change, *etc*. [44].

**Fig. (5):**
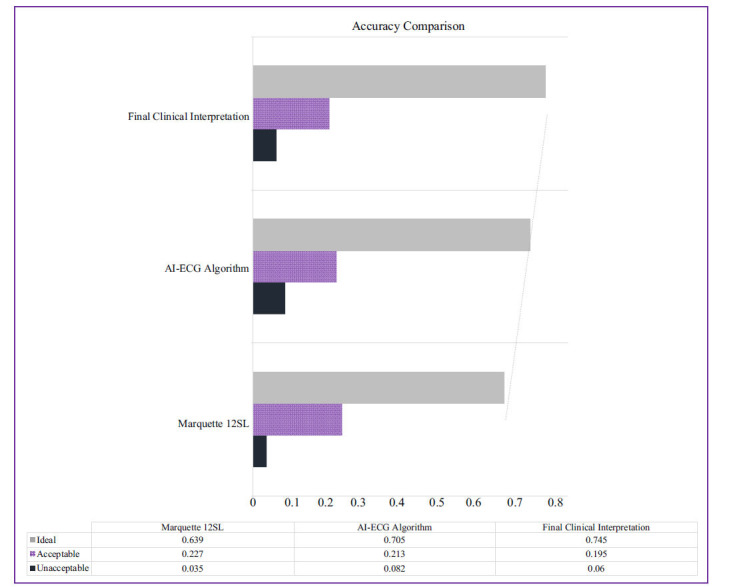
Comparing accuracy between the AI algorithm and computerized reader.

The results of the model were randomly compared on 500 ECGs with the computerized ECG reader, and clinical interpretations were then classified into 3 groups based on how satisfactory they were and the degree of further adjustments needed (Fig. **5**).

The algorithm outperformed the computerized and better-approximated expert over-reading for comprehensive 12-lead ECG interpretation.

A similar study done two years earlier in the same clinic developed an AI-enabled electrocardiograph using a CNN to detect the ECG signature of atrial fibrillation present during normal sinus rhythm (1 normal rhythm recorded). By using data from patients (180,922 individuals) from 1993 to 2017, the AF detected was verified and categorised by experts (Table **2**).

**Fig. (6):**
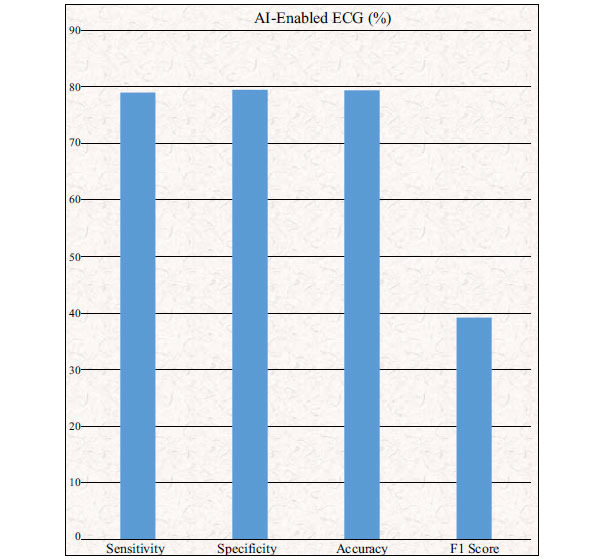
Showing results of the AI-enabled ECG.

The ECGs were allocated to the training, internal validation, and testing datasets in a 7:1:2 ratio. Before the normal sinus rhythm ECG was tested by the model, 3051 (8.4%) patients in the testing dataset had verified atrial fibrillation. The AI-enabled ECG identified atrial fibrillation with an AUC of 0.87 (95% CI 0.86-0.88), sensitivity of 79.0% (77.5-80.4), specificity of 79.5% (79.0-79.9), F1 score of 39.2% (38.1-40.3), and overall accuracy of 79.4% (79.0-79.9). However, including all ECGs acquired during the first month of each patient's window of interest (*i.e*., the study start date or 31 days before the first recorded atrial fibrillation ECG) increased the sensitivity, specificity, and accuracy (Fig. **6**) [45].

It was found that an AI-enabled ECG acquired during normal sinus rhythm permits identification at the point of care of individuals with atrial fibrillation.

## 6 RESULTS

In general, there has been notable effectiveness in sensitivity and accuracy across different neural networks in the past few years. The sensitivity ranged from approximately 80–100%, being especially high in the algorithm using deep belief networks from -10 to 5dB (96.4%- 100%) and a random sensitivity of 98.2%. The effectiveness of machine learning algorithms was marginally comparable to clinician detection (Table **3**).

## 7 COMPARISON OF DETECTION IN TRADITIONAL TECHNOLOGIES

Following are the results of comparing traditional technologies in terms of their detection ability:

- Paroxysmal AF is easily missed on usual ECG recordings.
- Results of 24-hour Holter recordings are not always fruitful.
- Implanted loop recorders find AF in less than 15% of patients in a year.
- The usage of clinical risk scores has shown limited power in predicting AF occurrence.

## 8 DISCUSSION

### 8.1 Discussion on Device-detected Atrial Fibrillation and Stroke Risk

Several studies have assessed the association between clinically and automated device-diagnosed AF and ischemic stroke [12, 46-50]. So far, there has been no clear distinction in the guidelines between atrial fibrillation diagnosed by either avenue and a difference in stroke risk. However, it is important to note that device-detected AF comprises implants in patients who already have established risk factors as well as more commercial hand-held devices in patients (ambulatory, asymptomatic, and symptomatic) who do not necessarily share the same risk factors or have as severe clinical presentations as the former. Therefore, it is prudent not to prematurely place artificial intelligence-calibrated devices on a prognostic pedestal because the clinical significance of AF detected in their usage might be less substantial in comparison to the significance in more advanced patients who were diagnosed or are already being managed by cardiologists [51-53]. According to different trials, AF burden, as well as clinical stroke risk (CHA2DS2-VASc scores), contributes to a patient’s risk for ischemic stroke. The result of the TRENDS study showed that stroke risk increased only when the burden of AF was greater than 5.5 hours, while a different study under 2-week monitoring suggested that the overall burden of AF, irrespective of the duration of the longest AF episode, was the most important determinant of ischemic stroke risk. This implies the significance of the accumulation of more patient data to better verify the prognosis, which AI technologies are endeavouring to potentiate [47, 54].

A new risk score known as the morphology-voltage-P-wave duration (MVP) is also being increasingly used as a prognostic marker for atrial fibrillation, particularly its recurrence. In 2021, an observational study by Mert İlker Hayıroğlu *et al.* was conducted on 266 participants, which were split into 3 score groups after acute ischemic stroke, and the prognostic reliability of the MVP score in assessing the chances of in-hospital and long-term AF diagnosis was investigated. Those who fell in the 2^nd^ group (5–6) developed atrial fibrillation at a significantly higher rate both in-hospital (13.2 times, 78% sensitivity, and 76% specificity) and in the long-term (5.2 times, 85% sensitivity, and 59% specificity). For long-term prediction, the sensitivity rate was markedly higher than the specificity, but it nonetheless bodes for some future usability in patient management decisions [63].

### 8.2 Discussion and Limitations in the Application of Machine Learning

As with any big-data technology, there are technical difficulties with the incorporation of AI algorithms into clinical practice. There are several variations in existing ECG input data types, as well as storage formats and forms of interpretation, that hinder the widespread interoperability of ECG data. These formats also vary depending on whether the data pertains to resting, ambulatory, bedside, or ECG from mobile devices [49]. This could cause miscommunication and discrepancies in the understanding of the data. Moreover, it is clear that AI algorithms need a large amount of high-quality data to provide accurate results, which is a major limitation in developing algorithms for the management of rare diseases with a limited sample size [55].

The majority of algorithms developed for the detection of AF rely on the recognition of absolutely irregular R-R intervals; they consequently have a high likelihood of missing cases of atrial flutter. Both arrhythmias confer a similar risk of thromboembolism, and therefore, their possible underdiagnoses by automated algorithms may undermine confidence in their reliability [56]. This is among other examples of diagnostic inaccuracy due to the technical modalities of how the algorithm works. Unfortunately, inexperienced physicians ordering the ECG may fail to identify mistakes in interpretation and accept the automated diagnosis without criticism. Clinical mismanagement may result in the risk of exposing patients to useless investigations or potentially dangerous treatment [57].

After the device suggests AF, the natural course is for the subjects to head to their respective healthcare providers, who would ideally conduct a qualified personnel-assessed ECG at the medical establishment and possibly continuous monitoring for a certain period. If the patient has a persistent form of AF, the condition is more likely to be confirmed by this method, and management commences. However, short episodes of AF are most likely to be missed, especially if there is a delay between the device detection and the patient’s presentation to the clinic or reassessment by qualified personnel. It creates the problem of not knowing if the normal ECG reading post-presentation to the clinic is because of device inaccuracy or the temporary nature of the episode. It does, however, have a role to play in monitoring the prognosis of AF in patients on a long-term basis. It is only tangible if the particular device used by the patient is backed by sufficient evidence to deem it reliable for such purposes. Other concerns include the high cost that would be incurred to implement widespread usage of ML devices by the population. There is also a degree of technological know-how needed for proper usage or application of the device to ensure accurate readings. This increases the possibility that the population actually targeted for AF screening (generally over 65 years of age) would be at a disadvantage because they would either not be able to afford it or most likely not be able to properly navigate the usage of such technologies [21, 58-61].

## CONCLUSION

To conclude, AF diagnosis has taken a positive picture due to the incorporation of modern algorithms in machine learning. In the controlled framework of some studies, the effectiveness of ML algorithms has been shown to supersede that of a physician in the detection of atrial fibrillation. Its incorporation into practice and portable devices promises to increase precision, timeliness, and, in other words, efficiency in the management of this and other arrhythmias. Nonetheless, the limit of its practicability in the populace remains, and improving the AF sensitivity, specificity, and accuracy is a gradual process that involves other influencing factors like patient population, differences in interpretation methods, the availability of technology, and the reliability of more commercial-grade (and less altruistically centered) technological options. This is, however, expected to improve in the coming years.

## Figures and Tables

**Table 1:** List of different machine learning methods.

**Supervised**	**Non Supervised**	**Semi-Supervised**	**Reinforcement**
Has known labels	Has no labels	Has labels for a smaller subset	A decision is made on previous experience (feed-back based)

**Table 2:** Showing the details of the dataset.

**Dataset**	**No. of Patients**	**ECGs Recorded**
Training	126,526	454,789
Internal validation	18,116	64,340

**Table 3:** Representing overall sensitivity and accuracy.

**Neural networks**	**DNN**	**DBN**	**CNN**
**Overall Sensitivity**	86.1%	73.4-100% (-5 to 10dB) 96.4-100% (-5 to 5dB)	79%
**Overall Accuracy**	AUC (91%)	75-99.5%	79.4%

## Data Availability

The data that support the findings of this study are openly available in • National Library for Biotechnological Information.NCBI at https://www.ncbi.nlm.nih.gov/pmc/articles/PMC6784839/ , doi:10.1038/s41591-018-0268-3 (reference number [5]). • *IEEE International Instrumentation and Measurement Technology Conference* at https://ieeexplore.ieee.org/document/7969948, 10.1109/I2MTC.2017. 7969948 (reference number [6]). • ScienceDirect at https://www.sciencedirect.com/science/article/pii/S2666693621000463, https://doi.org/10.1016/j.cvdhj.2021.04.002 (reference number [7]). • The Lancet journal at https://www.thelancet.com/journals/lancet/article/PIIS0140-6736(19)31721-0/ fulltext, https://doi.org/10.1016/S0140-6736(19)31721-0 (reference number [15]).

## LIST OF ABBREVIATIONS

AF: Atrial Fibrillation
ECG: Electrocardiogram
ESC: European Society of Cardiology
ML: Machine Learning

## SUPPLEMENTARY MATERIAL

PRISMA checklist is available as supplementary material on the publisher’s website along with the published article.
